# Emotional Intelligence and Good Medical Practice: Is There a Relationship?

**DOI:** 10.7759/cureus.23126

**Published:** 2022-03-13

**Authors:** Cameron Dott, George Mamarelis, Edward Karam, Kavyansh Bhan, Kash Akhtar

**Affiliations:** 1 Trauma & Orthopaedics, Royal London Hospital, London, GBR; 2 Trauma & Orthopaedics, Whipps Cross University Hospital, London, GBR

**Keywords:** medical education, psychology, good medical practice, surgical training, medical training, emotional intelligence

## Abstract

Emotional intelligence (EI) is defined as the ability to perceive and manage the emotions of oneself and others. Despite being one of the most highly used psychological terms in popular nomenclature, its understanding in the context of clinicians remains poor. There is a dearth of literature on this topic, and this submission examines the relationship between a clinicians’ EI and the key domains of “Good Medical Practice” guidelines from the General Medical Council, United Kingdom. It aims to review and critically analyse the existing literature on EI and Good Medical Practice while attempting to establish a relationship between the two.

This submission thus examines the relationship between emotional intelligence and a clinician’s on-the-job performance. The findings demonstrate how emotional intelligence can aid the clinician in all aspects of their working life in the context of practising in line with General Medical Council (GMC) guidance. The authors also recommend exploring the possibility of inclusion of EI within a modern medical curriculum, as it may lead to improved practice in clinicians.

## Introduction and background

Early definitions of emotional intelligence (EI) were suggested by Mayer and Salovey in 1990 and Goleman in 1995 [1-2]. They essentially defined EI as the ability to recognise and control one’s own and others’ emotions while utilising this awareness for self-control and influencing the emotions in other individuals [1-2]. In theory, therefore, an emotionally intelligent individual would possess the ability to acknowledge their feelings and emotions in a given scenario, remain level headed and decide upon the best course of action and in doing so have the capability to motivate other team members towards their goal. It is not surprising, therefore, that there is a general belief that emotionally intelligent individuals would be more successful in their endeavours in the workplace. Indeed, for that very reason, researchers have examined EI within the medical profession in order to attempt to elucidate whether EI is a key attribute within medical practice.

In the past three to four decades, the field of EI literature has expanded and the definition of EI evolved. The literature now recognises two distinct definitions of EI with separate ideologies. The first theory suggests that EI is a trait with the belief that it is more of an inherent quality in an individual’s character and personality measured through self-report methods while the second theory recognises EI as an ability and a skill that is measured through more objective means [3]. This distinction between a trait EI and an ability EI has also led to varying tools for its measurement.

In 2013, the General Medical Council (GMC) published updated guidance detailing the way in which doctors should practice. They named it the “Good Medical Practice” guidelines. Within this document, four key domains were highlighted, namely, Domain 1 - Knowledge, skills and performance; Domain 2 - Safety and quality; Domain 3 - Communication, partnership and teamwork; Domain 4 - Maintaining trust [4].

A review of the literature relating to EI between 1994 and 2018 was conducted and included articles that met the search criteria to the key domains within the GMC Good Medical Practice guidelines. The hypothesis was that higher levels of EI would correlate with improved performance in the key domains.

## Review

Methodology

Inclusion Criteria

Popular databases like MEDLINE, Embase, PubMed, American Psychology Association PsycINFO, and the Cochrane Database were searched for English language articles between 1994 and 2018. Terms used in the search included the following keywords appearing in either the title or abstract: ‘medicine’, ‘medical’, ‘surgery’, ‘surgical’, ‘clinical’, ‘physician’, ‘doctor’, ‘resident’, ‘trainee’ and ‘health care’. These were combined with the term ‘emotional intelligence’, which again needed to appear in the title or abstract.

Exclusion Criteria

Any article published in a language other than English were excluded from the review. Any review articles, commentaries, editorials or opinion pieces were also excluded. Articles studying EI in medical students or allied healthcare professionals were not included to maintain uniformity of the population being studied. Articles published after 2018 or before 1994 were not included.

**Figure 1:**
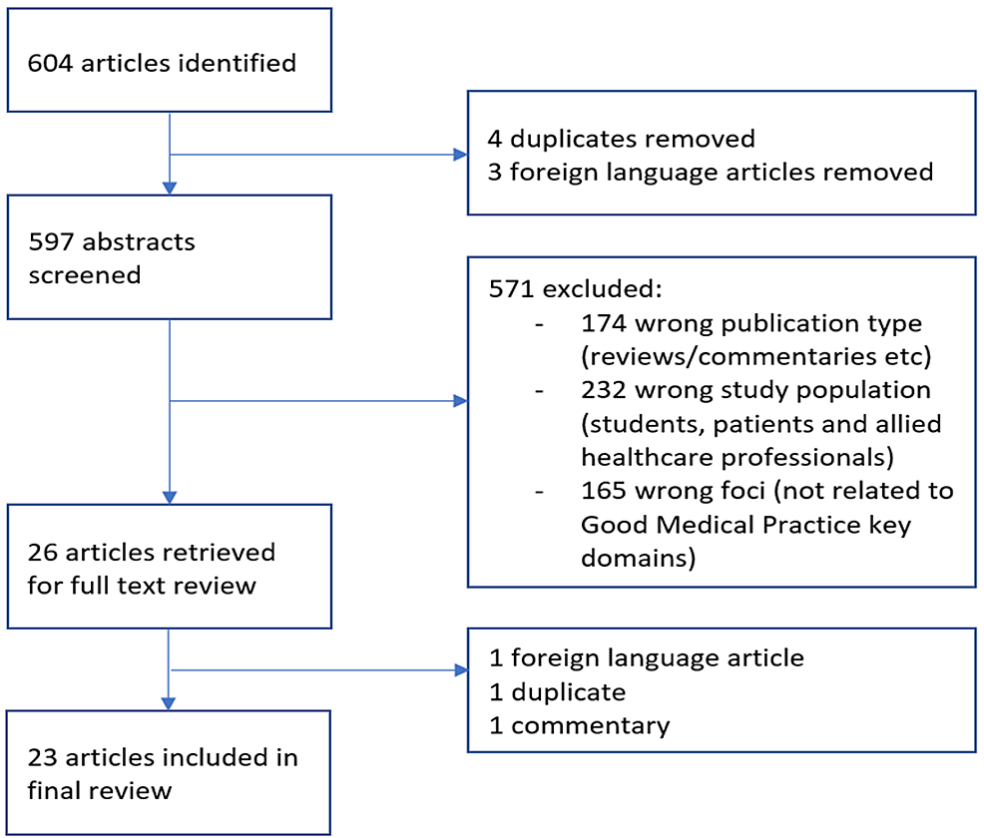
Flow diagram representing the process of inclusion/exclusion

The initial literature search identified 604 publications. Following abstract review, 26 full-text articles were retrieved and, of these, 23 were included in the final review. Below is a flow diagram representing the process of exclusion (Figure 1).

Review

Domain 1: Knowledge, Skills and Performance

It has been widely hypothesized that Emotional Intelligence provides an individual with indispensable skills like control of temperament, improved adaptability and self-management skills, which may lead to improved performances in high-intensity industries like healthcare. A higher EI may also enable individuals to learn new skills at a much faster rate [5]. In this submission, the authors reviewed eight articles that reported on associations between levels of EI in clinicians and its effects on their performance and skills [5-12]. Although two of the articles did not identify any positive correlation between overall levels of EI in residents and their performance, they found a slight negative correlation between certain aspects of EI and performance [10-11]. They found that performance in the Psychiatry Resident In-Training Exam (PRITE) and EI scores of the residents appearing for exams demonstrated an inverse relationship with each other [10]. Although surprising, since the general consensus amongst the population is that clinicians opting for mental health as a profession would demonstrate above-average emotional intelligence, the study results were limited by the small number of participants. Similarly, the article by Talarico et al. was a pilot study published initially in 2008 with a subsequent new publication in 2013 noting a significant positive correlation between EI in anaesthesiology residents and performance against the Accreditation Council for Graduate Medical Education (ACGME) core competencies in a much larger, multi-institution study [12]. With this new publication, the authors actually contradicted their own previously published work, although this may be attributed to the initial single site and limited participant study.

Four articles reported a positive correlation between the total EI score and some, but not all, of the chosen performance measures [5-7,9]. Higher levels of EI were seen to correlate with improved performance in the United States Medical Licensing Exam (USMLE) Steps 2 and 3 but not with Step 1 or American Board of Surgery In-Training Exam (ABSITE) score [6-7]. This may be attributed to the fact that USMLE Step 1 and ABSITE exams test domains are beyond the scope of more traditional USMLE Step 2 and Step 3 exams [6]. Lin et al. also looked at the level of EI in applicants to a general surgery residency and found that higher EI was associated with improved interview ranking [7]. Gardner et al. found higher EI correlated with significantly improved performance in situational judgement tests in general surgery residents although there was no such correlation seen with overall performance on the residency programme [5]. Another study published in 2009 compared the EI levels of Internal Medicine residents at the start and end of the academic year and found that an improvement in EI along the course of the academic year directly correlated with improved clinical performance and lower burnout amongst the residents [9]. This suggested that EI may have a significant clinical impact on the overall performance and wellbeing of residents. Although it may be argued that residents mature with time and experience, the possibility of EI playing a role in this improvement cannot be entirely ruled out.

Another study by Park et al. found a positive correlation between higher levels of emotionality (a facet of global EI as measured by a self-assessment tool) and competency progression in otolaryngology residents [8]. It was suggested that personal characteristics may affect the resident progression towards competency improvement, with higher EI being directly proportional to higher competency achievement. Thus, the authors recommended providing the residents with opportunities to actively engage in activities that provide for personal and professional development to improve their overall clinical and professional progression [8].

Domain 2: Safety and Quality

In the interest of patient safety, the GMC Good Medical Practice guidelines require that all individual clinicians perform safely in everyday duties [13]. Two articles that satisfied the inclusion criteria relating to this domain of the GMC were reviewed and critically analysed [13-14]. De Maores et al. measured EI scores in critical care trainees and correlated them to their self-awareness [13]. They found that individuals with higher EI also had higher levels of self-awareness. This self-awareness may assist clinicians in recognising their limitations and make them safe doctors. Recognising the point at which a senior should take over is one of the key elements of resident training [13]. This allows the residents to understand the importance of teamwork and enables continuous medical education and upskilling. Moreover, clinicians with a higher EI were also found to have a lower rate of burnout and improved job satisfaction. This directly correlates with improved performance at the job, diminishing the possibility of making errors due to stress, anxiety and improper rest [14].

Vafaei et al. explored the relationship between levels of EI and aspects of human resources risk among staff working in emergency wards in two hospitals [14]. Whilst they appear to suggest that higher EI relates to lower levels of behavioural, occupational health and knowledge and skills risks, it is very difficult to interpret their results due primarily to a lack of transparency/detail regarding their methodology, in particular relating to the measurement tool used for EI. Secondly, the selection criteria for the study population are unclear, which makes direct comparisons difficult.

Domain 3: Communication, Partnership and Teamwork

A total of nine articles related EI to this domain [13,15-22]. Seven out of the nine articles reported a positive correlation of communication, partnership and teamwork with the respective EI levels. While four of these articles used patient satisfaction as a measure associated with physician EI levels, only two detailed positive correlations [16,22]. Dugan et al. showed that an improvement in the EI scores of otolaryngology residents achieved through a carefully designed training programme directly correlated with improvement in patient satisfaction scores of these residents [16]. The other two articles, however, reported both a positive and no correlation between EI level and patient satisfaction [19,21]. Wagner et al. found no effect of higher global EI levels on patient satisfaction but did find that higher levels of happiness (a subscale of their measurement tool of EI) related to improved patient satisfaction [19]. Weng et al. found improved patient satisfaction with greater physician EI reported at the initial patient visit but no such correlation was seen at their two-week follow up [21]. They suggested that a higher EI score had a positive effect pre-surgery on both patient satisfaction and patient-surgeon relationship, with this effect diminishing on the post-operative follow-up. However, Weng et al. did acknowledge a possible limitation of their study in the form of selection bias since their study relied on patients completing a questionnaire, and it was suggested that ‘satisfied’ patients are more likely to complete a questionnaire than ‘unsatisfied’ patients [21].

Two articles included in this review found no positive correlations between EI and the key domain of communication, partnership and teamwork [18,20]. One examined the relationship between EI and breaking bad news skills and found that higher EI alone was not sufficient enough to effectively deliver the news of death to patients [18]. They found that no aspect of EI correlated with the death notification protocol followed at the authors' institution, suggesting that the highly complex nature of the task of delivering bad news meant that breaking bad news needed much more than the clinician’s ability to recognise and regulate emotion [18]. Weng et al. assessed patient satisfaction following outpatient consultation and compared this to the self-rated EI of the physicians [20]. They argued that owing to the multi-dimensional nature of EI, it was difficult to attribute study findings to EI alone. They recommended a further refinement of the definition of EI to allow an assessment of the validity of EI and its effects on clinician and patient-related outcomes.

A further two articles discussed the effect of EI on communication skills with both reporting improved communication in physicians with higher levels of EI [13,15]. Cherry et al. noted that clinicians with high EI tend to enquire about patient emotion at a more appropriate time as compared to clinicians with a low EI [15]. They also suggested that clinicians with high EI also recognise when the patients do not need to be enquired about emotion, and thus pick up emotional cues much more effectively as compared to clinicians with a poor EI [15]. Moreover, Faye et al. in their study of Indian Postgraduate Medical residents found that higher levels of self-control (a facet of EI) were associated with improved relationships with colleagues and improved participation in teamwork [17].

Domain 4: Maintaining Trust

Five articles examined the relationship between a clinician's EI and maintaining patient trust [23-27]. However, two examined a mixed population of health care workers, which included both physicians and nurses [23-24]. Dafeeah et al. reported improved attitudes towards patients with human immunodeficiency virus acquired immunodeficiency syndrome (HIV/AIDS) with higher physician EI [23]. However, the study was based on subjective self-reports of the healthcare professionals themselves, creating a high possibility of bias. Moreover, the authors acknowledge the fact that the majority of the population included in the study consists of expatriate healthcare workers of Qatar who may be forced to provide treatment to patients regardless of their HIV/AIDS status owing to lack of job security in the country [23]. Deshpande in 2009 reported that a higher EI was associated with improved ethical behaviour [24]. However, in view of the fact that these studies had a mixed sample population, conclusions should be drawn with caution.

**Figure 2:**
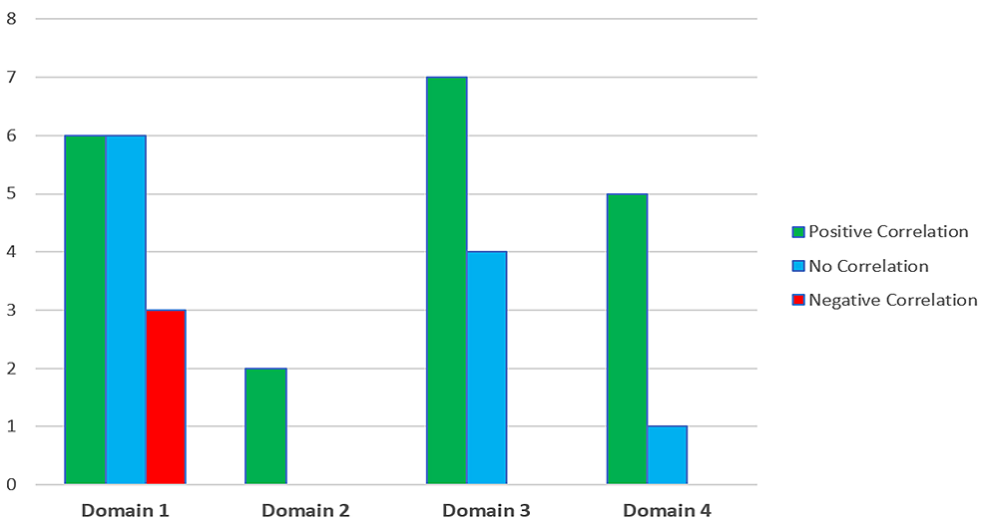
Total number of articles reviewed for each key domain

The remaining three articles were all from the same lead author and examined the effect of a doctor’s EI on patient trust [25-27]. In the first study, the authors found no correlation between a doctor’s self-rated EI and the level of patient trust in the outpatient environment [25]. However, there was a significant positive correlation seen between nurse ratings of a doctor’s EI and the level of trust in the same patients. The subsequent, larger studies used nursing directors to assess each doctor’s EI levels and both found statistically significant correlations between higher measurements of EI and levels of patient trust [26-27]. Figure 2 depicts the total articles reviewed for each domain.

Discussion

To our knowledge, this is one of the first review articles to examine the relationship between EI and a doctor’s on-the-job performance by benchmarking medical practice guidelines. A few systematic reviews have examined the role of EI in medicine but with a focus on medical and surgical education [28-29]. Cook et al., in their systematic review, focused on the influence of EI on success in medical school admissions and programme matriculation [28]. They concluded that EI was only associated with some measures of success in matriculation but with none of the measures used for admission [28]. Another systematic review by Arora et al. examined EI in the context of ACGME competencies [29]. They found a positive association between EI and many of the ACGME competencies and suggested that EI-related skills be incorporated into medical training curricula to help students achieve key competencies [29].

Overall, the findings reported in this review are suggestive of the fact that a doctor with a higher level of EI may be better suited to practising in line with the GMC Good Medical Practice guidance. Only three studies found a negative correlation with EI [7,10,11]. Interestingly, all three studies related to the first domain of the GMC guidance (knowledge, skills and performance). Lin et al. sought to determine the effect of EI levels in residents on their performance in interviews [7] while Schrimpf et al. evaluated its effects on Psychiatry Resident In-Training Exams (PRITE) [10]. Talarico et al. evaluated the anaesthetic resident’s performance as assessed by faculty on the resident programme [11]. Of note, the latter two studies only identified a negative correlation between EI and performance when they analysed the various subscales of EI as measured by the Bar-On Emotional Intelligence Quotient with no correlation found when using residents’ global EI scores [10-11].

Limitations

There are two prime limitations of this study. Firstly, the framework provided by the GMC, whilst effective guidance, does not necessarily provide an exhaustive list of all aspects of good medical practice. There may therefore be skills or qualities not covered within the guidance and hence do not feature in this review.

The second limitation relates to the measurement of EI. There are several different measurement tools used for EI - we identified eight in this article (Bar-On Emotional Quotient Inventory, Mayer-Salovey-Caruso EI Test, EI Appraisal, Wong and Law EI Scale, Emotional Quotient Self-Assessment Checklist, Trait EI Questionnaire, Emotional and Social Competence Inventory, EI Survey) [30-37]. Drawing conclusions from separate studies is therefore challenging. A good example of the potential challenges with EI measurement comes from one of the papers by Weng et al. [25]. They used the self-assessment Weng and Law EI Scale (WLEIS) to measure EI scores in clinicians and had nurses assess clinicians’ EI using the WLEIS tool. This produced contradictory findings when they examined the effect of a doctor’s EI on patient trust with there being no correlation between a doctor’s self-rated EI score and patient trust but a positive correlation between nurse-rated EI in doctors and the level of patient trust. The issue with varying EI measurement across different studies and its effect on drawing conclusions also features as a common finding in the other systematic reviews mentioned above [28-29].

## Conclusions

Whilst there are certainly flaws within the literature relating to EI, it does seem that there is evidence supporting a positive role for EI within medical practice. It has been suggested in the literature that a higher EI plays a positive role in all four aspects of Good Medical Practice, namely, knowledge, performance, safety and trust. Moreover, the key components of a clinician's daily practice involve aspects that are heavily affected by their EI, namely, teamwork, communication with both patients and colleagues, building trust with patients and overall clinical performance of an individual clinician. Recognising that EI impacts these key domains of an individual's practice and taking steps to improve the EI may be advocated. It is clear from the review that a definite relationship exists between EI and the key domains of Good Medical Practice. Incorporating EI into a holistic medical education would therefore seem sensible, particularly given the evidence that EI levels can increase through focused education. A key issue remains the measurement of EI and future focus should therefore be on exploring this further, perhaps with the aim to standardise EI measurement to allow more conclusions to be drawn from various studies.
